# Signaling Mechanisms in the Regulation of Renal Matrix Metabolism in Diabetes

**DOI:** 10.1155/2012/749812

**Published:** 2012-02-19

**Authors:** Meenalakshmi M. Mariappan

**Affiliations:** Division of Nephrology, Department of Medicine, MC7882, South Texas Veterans Healthcare System, University of Texas Health Science Center, 7703 Floyd Curl Drive, San Antonio, TX 78229-3900, USA

## Abstract

Renal hypertrophy and accumulation of extracellular matrix proteins are among cardinal manifestations of diabetic nephropathy. TGF beta system has been implicated in the pathogenesis of these manifestations. Among signaling pathways activated in the kidney in diabetes, mTOR- (mammalian target of rapamycin-)regulated pathways are pivotal in orchestrating high glucose-induced production of ECM proteins leading to functional and structural changes in the kidney culminating in adverse outcomes. Understanding signaling pathways that influence individual matrix protein expression could lead to the development of new interventional strategies. This paper will highlight some of the diverse components of the signaling network stimulated by hyperglycemia with an emphasis on extracellular matrix protein metabolism in the kidney in diabetes.

## 1. Introduction

The importance of hyperglycemia in renal injury was confirmed by the Diabetes Control and Complications Trial [1] and the United Kingdom Prospective Diabetes Study [2], which demonstrated that diabetic kidney disease can be prevented by keeping blood sugar in target range; however, this is difficult to achieve. Diabetes, particularly type 2, is the most common cause of end-stage renal disease requiring chronic renal replacement therapy in the US. Despite its high prevalence, the mechanism of development and progression of diabetic nephropathy (DN) is still not fully understood, partly because of unrecognized and undiagnosed kidney changes that coexist during latent diabetes [3]. Much of our understanding of the mechanisms of injury in diabetes comes from studies on rodent models of diabetes. Although several such animal models of diabetes exist, no single animal model develops renal changes identical to those seen in humans. Brosius et al. [4] have compiled a report on the progress towards establishing and validating a murine model of human DN (http://www.diacomp.org/). It is likely to be difficult to generate a single mouse model that recapitulates all of the features of human DN.

Pathophysiology of DN involves an interaction of genetic, metabolic, and hemodynamic factors. Structural renal changes in diabetes start with glomerular hypertrophy, followed by glomerular basement membrane (GBM) thickening, mesangial matrix expansion, and development of sclerotic lesions [5]. Accumulation of extracellular matrix proteins is governed by a balance between increased synthesis regulated at the level of transcription and mRNA translation, and, degradation regulated by such processes as the balance between proteolytic activity of matrix metalloproteinases (MMPs) and their inhibitors, the tissue inhibitor of metalloproteinases (TIMPs). A variety of growth factors and cytokines participate in this pathology through complex signal transduction pathways in a cell-specific manner. In this review we will discuss the various mechanisms by which hyperglycemia can induce extracellular matrix synthesis and accumulation in diabetic kidneys.

## 2. Extracellular Matrix Components

An early sign of renal involvement in diabetes is an increase in basement membrane thickness that has been described as a prediabetic lesion [6]. The thickening of the renal basement membranes of the glomerulus (GBM) and tubules (TBM) is due to a consequence of the hyperglycemia-induced metabolic perturbations resulting in augmented synthesis and accumulation of intrinsic ECM components at these sites [7, 8]. The major TBM components are type IV collagen, laminin, and entactin while collagen types I, V, and VI and fibronectin are generally considered components of the renal interstitium. The GBM is predominantly composed of laminin, collagen type IV alpha 3 to alpha 5, agrin, and perlecan. In diabetes it has been shown that collagen *α*3 through  *α*5 (IV) chains, collagen V, laminin, fibronectin, and serum proteins contribute to thickened GBM [9]. Mesangial expansion is largely due to the accumulation of extracellular matrix (ECM) proteins such as collagen  *α*1, *α*2 (IV) chains, collagens V and VI, laminin, and fibronectin [10, 11].

### 2.1. Laminins

Laminins are glycoproteins expressed primarily in basement membrane. Laminins are heterotrimeric structures consisting of combinations of five alpha, three beta, and three gamma chains that share a common domain and several globular and rod-like domains. The tissue-specific distribution of laminins is mainly determined by expression of the alpha chains; in particular, a number of alpha 5-chain mutations are associated with neonatal lethality and defective glomerulogenesis [12, 13]. Glomerular and proximal tubular epithelial cell laminin expression has been shown to increase in response to hyperglycemia and TGF-b coincident with increased thickening of the glomerular basement membrane [14, 15].

### 2.2. Collagen Type IV

Collagens serve as fibrotic markers in diabetic nephropathy, and type IV collagen provides the basic structural framework of the glomerular ECM. There are six genetically distinct alpha chains (*α*1 through  *α*6), and all have similar domain structures. In the GBM alpha 3 through alpha 5 predominate whereas in the TBM alpha 1, 2, 3, and 5 are present [16, 17].

### 2.3. Fibronectin

Fibronectin is a glycoprotein that is found in the plasma as well as in the basement membrane and the mesangium of the glomerulus [18, 19]. Types V and VI collagen along with type IV, and fibronectin colocalize in a similar distribution in the glomerular subendothelial area and the mesangium. Entactin/nidogen (En/Nd) is an elongated approximately 150 kDa molecule containing three globular domains separated by two linear segments. Laminin, type IV collagen, and fibronectin are all capable of self-aggregation. Laminin also has additional binding sites for glycosaminoglycans and attaches to collagen via nidogen bridge. It serves as a link between the laminin and collagen IV networks in sub-endothelial, subepithelial and mesangial areas in the glomerular basement membranes [20, 21]. The networking pattern of these ECM components determine the pore size and the charge-selective properties of GBM [22].

## 3. Role of Transcription, mRNA Translation, and Ribosome Biogenesis in ECM Production

### 3.1. Transcription

Regulation of protein synthesis may occur at the level of transcription or mRNA translation. High glucose stimulates the transcription of matrix genes and represses matrix degradation leading to glomerulosclerosis [23–25]. Sanchez and Sharma [26] have extensively reviewed the mechanism of activation of transcription factors involved in the progression of diabetic kidney disease including upstream stimulatory factors (USF1 and 2), activator protein 1 (AP-1), cAMP-response element-binding protein (CREB), nuclear factor (NF)-*κ*B, nuclear factor of activated T cells (NFAT), and stimulating protein 1 (Sp1). Until the beginning of the last decade transcription was the only studied regulatory mechanism for increased ECM protein synthesis induced by hyperglycemia in the renal tissues. Accumulating evidence from recent studies has established mRNA translation as another important and independent step in the regulation of protein synthesis [27–30].

### 3.2. mRNA Translation

The process of mRNA translation occurs in three steps: the initiation phase, which involves localization of the preinitiation complex containing the 40S ribosomal subunit and the initiator methionyl tRNA to the AUG (methionine) codon on the mRNA; the elongation phase, during which amino acids are added to the nascent peptide according the codon sequence of the mRNA; the termination phase, in which arrival at a stop codon leads to the release of the completed peptide chain. Of these three steps, initiation is the rate limiting step as it determines the recruitment of ribosomes to the specific mRNA. Elongation itself is composed of three traditionally defined steps: eEF1A-directed binding of the aminoacyl-tRNA to the A site (aminoacyl site) of the ribosome, peptide bond formation triggered by the enzymatic activity of the ribosome (the peptidyl transferase center), and eEF2- mediated translocation which moves the peptidyl-tRNA from the A site to the P site (peptidyl site) by precisely one codon (three nucleotides) [31–33]. Upregulation of these events result in augmented translational efficiency. Signaling pathways play a major role in regulation of translation. Among them the mTOR system regulates the initiation and elongation phases of translation of specific mRNAs to culminate in increased protein synthesis [31–33]. Such control is generally exerted through changes in the phosphorylation states of the translation initiation or elongation factors. Diabetic kidney tissues and renal cells treated with high glucose demonstrate activation of various initiation and elongation factors that are involved in regulation of mRNA translation [14, 15, 34]. Activation of these factors by high glucose and angiotensin II resulted in upregulation of selective proteins like laminin beta 1 chain and vascular endothelial growth factor (VEGF), respectively, in tubular epithelial cells [15, 35].

### 3.3. Ribosome Biogenesis

Ribosome biogenesis is a complex well-coordinated process in which hundreds of different proteins interact in the folding and processing of ribosomal RNA (rRNA) consisting of a small (40S) and large (80S) subunit in eukaryotic cells. The large subunit is composed of 5S, 28S, 5.8S rRNAs whereas the 40S subunit contains 18S rRNA. Furthermore, approximately 80 different ribosomal proteins (r-proteins) are found in eukaryotic ribosomes [36]. Ribosomes consisting of 80S and 40S ribosomal subunits and ribosomal proteins are part of the translation machinery that aid in carrying out the process of peptide synthesis by the addition of amino acids through translation of the genetic code in mRNA. The smaller (40S) subunit of the ribosome serves as a platform to bring together messenger RNA, aminoacylated transfer RNAs, and translation factors. The larger (80S) ribosomal subunit provides peptidyl transferase activity to catalyze peptide bond formation in nascent polypeptides. Increased production of ribosome reflects enhanced capacity for translation. Ribosome biogenesis is so important for cell growth that a growing yeast cell synthesizes approximately 2000 ribosomes every minute, requiring 60% of total cellular transcription. In mammalian cells [37], this number is even higher; for example, a HeLa cell makes 7500 ribosomal subunits per minute [38]. Ribosome biogenesis is regulated by the activity of RNA polymerase I, which controls the rate of rRNA synthesis. The activity of RNA polymerase I at the ribosomal DNA promoter is modulated by a complex of proteins, which includes the nucleolar protein upstream binding factor (UBF) 1. UBF1 interacts with the protein complex TIF-1B (SL1 in humans), which consists of the TATA box-binding protein and three associated factors. The resulting complex promotes the binding of RNA polymerase I to the ribosomal DNA promoter [39, 40]. The activity of UBF1 is regulated, at least in part, by its phosphorylation at Serine 388 [41]. We observed increased UBF phosphorylation at Ser388 accompanied by increased rDNA transcription in glomerular epithelial cells treated with high glucose and in kidney tissues from type 2 diabetic mice model [42]. This eventually leads to increased rRNA molecules and ribosomal proteins thereby increasing translational capacity and sets the stage for increased matrix protein synthesis in renal tissues and cells in response to hyperglycemia.

## 4. Signaling Pathways Activators and Inhibitors of Protein Synthesis

### 4.1. mTOR

**Figure 1:**
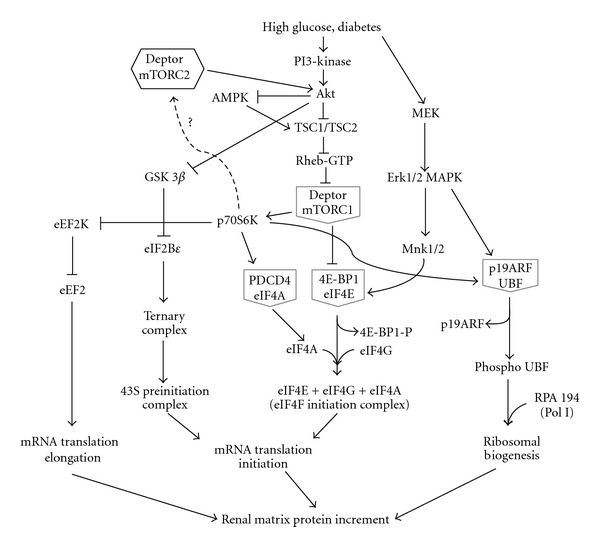
Intracellular signaling cascades regulated by high glucose leading to activation of promoters and suppression of intrinsic inhibitors of protein synthesis. Grey pentagons show the positive regulators held in an inactive repressor complex with an inhibitory protein. mTORC2 role in high glucose-induced protein synthesis has to be determined.

At the molecular level, mTOR is recognized as the mediator of signals from extracellular high glucose milieu to the nuclear contents of the cell. The complex signaling cascade regulated by high glucose to induce extracellular matrix protein synthesis is summarized in Figure 1. mTOR exists in two distinct physical and functional complexes, namely, mTORC1 and mTORC2 [43]. mTORC1 comprises mTOR, raptor, and mLST8; it phosphorylates the translation initiation regulators, p70S6 kinase and 4E-BP1, resulting in the changes in the activity of a number of initiation and elongation factors [44, 45]. In the resting cell, eukaryotic initiation factor 4E (eIF4E) is held inactive by its binding protein, 4E-BP1 [46]. When a stimulus for protein synthesis is received, mTORC1 is activated and it phosphorylates 4E-BP1. Phosphorylation of 4E-BP1 results in dissociation of eIF4E-4E-BP1 complex and release of eIF4E which then binds to the cap of the mRNA [47–50]. This augments the efficiency of translation. Phosphorylation of p70S6 kinase by mTORC1 affects both the initiation and elongation phases of mRNA translation. Activated p70S6 kinase phosphorylates ribosomal proteins and regulates ribosomal function. It also phosphorylates eukaryotic elongation factor 2 kinase (eEF2 kinase) which inhibits its activity [51, 52]. Decreased activity of eEF2 kinase contributes to reduced phosphorylation of eEF2 which results in activation of the latter [53]. As mentioned above, activated eEF2 facilitates the movement of aminoacyl tRNA from the A site to the P site on the ribosome during elongation phase of translation [51]. Thus, activation of p70S6 kinase facilitates the addition of amino acids to the newly synthesized peptide. Kidney tissues from type 2 diabetic *db/db* mice showed activation of mTORC1 that coincides with renal hypertrophy and matrix expansion. The constituents of the mesangial matrix expansion in the *db/db *mouse kidney consist of increased type IV collagen, fibronectin, and laminin [54, 55]. We have reported increase in laminin content in glomeruli and tubules by immunohistochemistry and morphometry in *db/db* mice kidneys when compared to *db/m* control mice; these diabetes-associated changes were inhibited by rapamycin. Ameliorative effect of rapamycin was shown to be due to inhibition of mTORC1 and its downstream pathways regulating the elongation phase of mRNA translation [34].

The mTORC2 complex contains mTOR, rictor, SIN1, and mLST8. Recent work has revealed that it controls the phosphorylation of the antiapoptotic proteins Akt/PKB and serum and glucocorticoid inducible kinase (SGK) and may promote cell survival [56–58]. Translation and processing of nascent polypeptides are highly coupled events that result in the production of mature and functional proteins. Recent investigations show that while mTORC2 activation of Akt and SGK1 can modulate translation, this complex also becomes recruited to the translating ribosome in order to process the newly synthesized polypeptide [59, 60]. The ribosome serves as a platform for cotranslational processing, folding, and transporting proteins to their target sites [61].

Most studies so far have been based on pharmacological inhibition of mTORC1 by rapamycin. Systemic administration of rapamycin, a specific and potent inhibitor of mTORC1, ameliorated pathological changes and renal dysfunction in diabetes [14, 62–65]. Thus, inactivation of mTORC1 is protective and reduced the effect of Erk- and TGF-beta-mediated prosclerotic pathways. However, cell-specific role of mTOR in renal hypertrophy induced by high glucose remained to be explored. Recent work by G$\ddot{\text{o}}$del et al. [66] and Inoki et al. [67] shows that genetic reduction of mTORC1 activity by eliminating 1 *Raptor *allele prevents podocyte injury and ameliorates the progression of common glomerular diseases such as diabetic nephropathy; mTORC1 activation induced by ablation of an upstream negative regulator Tsc1 recapitulated many DN features, including podocyte loss, glomerular basement membrane thickening, mesangial expansion, and proteinuria in nondiabetic mice. Thus, mTORC1 remains an attractive target for potential therapeutic target to prevent DN. 

Increase in protein synthesis occurs not only by stimulation of transcription and translation by also by inhibition of molecules that inhibit these processes. Signaling mechanisms augmenting protein synthesis have received much attention [14, 34, 62, 68, 69]. In contrast, constitutive signaling mechanisms that counteract the prohypertrophic signaling mechanisms and inhibit protein synthesis are not well understood. There are at least three important constitutive inhibitors of protein synthesis. They are AMP-activated protein kinase (AMPK) and Deptor that inhibit the activity of mTOR and glycogen synthase kinase 3 beta (GSK 3*β*) that inhibits the activity of eukaryotic initiation factor 2B epsilon (eIF2B*ε*).

### 4.2. AMPK

AMPK plays a dual role in cell metabolism. It serves as an energy sensor and as a part of AMPK-TSC pathway, it inhibits Rheb/mTORC1 and keeps protein synthesis in check. Hyperglycemia reduced AMPK activity by phosphorylation at Thr172 on the catalytic alpha subunit resulting in activation of mTORC1 in glomerular epithelial cells [14]. Activation of mTORC1 contributes to the renal changes characteristic of DN, including glomerular hypertrophy, glomerular basement membrane (GBM) thickening, and the accumulation of mesangial matrix [14, 70–72]. Inhibition of AMPK by high glucose is required for high glucose-induced hypertrophy and ECM protein increment. Treatment of neonatal rat cardiomyocytes and renal glomerular epithelial cells with metformin, AICAR, or resveratrol activated AMPK and inhibited the development of hypertrophy induced by agents such as high glucose or phenylephrine [14, 71, 73]. Thus, AMPK could be a potential target for intervention in diabetic nephropathy.

### 4.3. GSK 3*β*

GSK 3*β* is a ubiquitously expressed, highly conserved serine/threonine protein kinase found in all eukaryotes. Unlike most protein kinases involved in signaling, GSK 3*β* is active in unstimulated, resting cells and it is inactivated upon phosphorylation at Serine 9. GSK 3*β* is inactivated during hypertrophy of skeletal myotube [74], heart [75, 76] and pulmonary artery smooth muscle [77]. GSK 3*β* phosphorylates its substrate eIF2B epsilon in the resting cell [78]. Activity of eIF2B epsilon is important for the formation of the preinitiation complex during the initiation phase of mRNA translation [79]. We observed that GSK 3*β* inhibits high glucose-induced protein synthesis in renal proximal tubular epithelial cells and renal tissues by inhibiting the activity of eIF2B*ε* [80]. Type 2 diabetic *db/db* mice showed increased phosphorylation of renal cortical GSK 3*β* and decreased phosphorylation of eIF2B*ε*, which correlated with renal hypertrophy at 2 weeks, and increased laminin *β*1 and fibronectin protein content at 2 months. These data raise the possibility that renal hypertrophy and laminin *β*1 accumulation induced by type 2 diabetes could be rescued by the activation of GSK 3*β* or by overexpression of an active form of GSK 3*β* in the kidney in the *db/db* mouse. In transgenic mice overexpressing activate form of GSK 3*β* in the heart, the hypertrophic response to calcineurin activation was severely impaired [81]. However, it is interesting to note that transgenic mice overexpressing constitutively active form of GSK 3 (GSK3^S9A/S21A^ knockin mice) exhibit glomerular injury with proteinuria [82].

### 4.4. Emerging Target in Signaling

Deptor (DEPDC6-DEP domain-containing and mTOR-interactive protein) is a novel mTOR regulatory protein that interacts with mTOR in both mTORC1 and mTORC2 and negatively regulates mTOR activity [83, 84]. As discussed earlier, mTORC1 is a central regulator of protein synthesis, ribosome biogenesis and cell growth during diabetic kidney. A series of elegant experiments based on loss-of-function strategy by Peterson et al., [85] showed that Deptor interacts directly with both mTOR complexes and inhibits downstream pathways regulated by both complexes. Liu et al., [86] have shown that enhanced interaction between mTOR and Deptor by resveratrol, a known inhibitor of mTORC1 [70], negatively regulated leucine-induced mTORC1 signaling in C2C12 myoblasts. Finally Deptor knockdown in vivo largely prevented the atrophic response produced by immobilization and, in part, this response was mediated by an increased muscle protein synthesis [87]. Given its powerful role as mTOR regulator, investigating the role of Deptor in diabetic kidney disease may provide a new avenue for preventing renal matrix accumulation.

## 5. Micro RNA (miR) in ECM Synthesis and Accumulation

miR microarray identified five miRs (192, 194, 204, 215, and 216) that were highly expressed in human and mouse kidney [88]. A seminal report by Kato et al. demonstrated that the expression of miR 192, one of the highly expressed miRs in the mouse kidney, is increased in the glomeruli from *db/db* type 2 diabetic mice when compared to control mice [89, 90] and in mesangial cells treated with high glucose [91]. Upregulated expression of miR 192 occurs as a consequence of TGF beta increment in diabetic glomeruli which in turn increased the expression of Col1a2. Wang et al. [91] demonstrated that in cultured human MCs exposed to high glucose or TGF-beta, as well as in mouse DN models in vivo, there was a significant upregulation of miR-377 that indirectly led to enhanced fibronectin production. Long et al. [92] identified miR-29c expression in the kidney glomeruli obtained from *db/db* type 2 diabetic mice in vivo and in kidney microvascular endothelial cells and podocytes treated with high glucose in vitro that has been found to enhance ECM protein accumulation.

Recent studies have elucidated the role of miRs in controlling translation [93]. miRs regulate gene expression by inhibiting translation and/or by inducing degradation of target messenger RNAs [94]. miRs bind directly to 3′ untranslated regions of specific transcripts and most often directly repress translation; furthermore, an mRNA can be simultaneously repressed by more than one miRNA species or one miRNA can modulate more than one transcript [95]. Programmed cell death 4 (PDCD4), an endogenous inhibitor of translation, has been identified as a target of miR 21, and it will be interesting to investigate the role of miR 21 in regulating ECM protein synthesis induced by diabetes. Dey et al. [96] reported that high glucose and TGF*β* increase miR-21 and miR-214 in mesangial and proximal tubular epithelial cells. These microRNAs target downregulation of PTEN, an endogenous inhibitor of PI3 kinase dependent downstream Akt activity, for translational repression. The field of miRs and their role in diabetic kidney disease are an emerging field of investigation, and further studies will unravel the regulatory mechanism of these so-called “junk” DNA sequences in the genetic code [97].

## 6. Epigenetic Modification in ECM Production

Epigenetics is defined as mechanisms that affect chromatin structure and gene expression and dysregulation of the epigenome can also lead to disease. Major pathologic mediators of diabetes such as hyperglycemia, inflammatory factors, cytokines, and growth factors can lead to dysregulation of epigenetics [98]. Epigenetic changes include DNA methylation (covalent attachment of methyl groups at CpG dinucleotides), histone modifications (acetylation, methylation, phosphorylation, and ubiquitination), and RNA-based silencing. In the context of diabetic nephropathy, the altered state of the epigenome may be the underlying mechanism contributing to a “metabolic memory” that results in chronic inflammation and vascular dysfunction in diabetes even after achieving glycaemic control [99–101]. Identification of genetic and epigenetic risk factors that modulate ECM protein expression individually could provide the basis for the development of novel treatments and newer animal models of diabetic nephropathy.

## 7. Renal ECM Metabolism in Animal Models of Type 2 Diabetes

A higher proportion of individuals with type 2 diabetes are found to have microalbuminuria and overt nephropathy shortly after the diagnosis of their diabetes, because diabetes is actually present for many years before the diagnosis. This is why animal models of type 2 diabetes are very important so that newer and specific markers of early kidney injury could be identified before clinical diagnosis of the disease. However, these animals could only recapitulate some of the features of diabetic kidney disease seen in humans [102, 103]. T2DM is a complex genetic disease comprising many metabolic disorders with a common phenotype of glucose intolerance. Cohen et al. [104] have documented that glomerular pathology in type 2 diabetic *db/db* mice is accompanied by definable alterations in renal function, which are similar in chronology and nature to those found in human diabetes. Studies in *db/db* mice with type 2 diabetes have shown that accumulation of the renal matrix protein laminin-beta 1 is not associated with increase in its mRNA, suggesting potential regulation by mRNA translation [54]. Since hyperglycemia is associated with hyperinsulinemia, coinciding with the onset of laminin accumulation in the kidney in *db/db *mice, augmented laminin mRNA translation could be due to either elevated glucose or high insulin. If hyperinsulinemia was to be implicated in laminin regulation in type 2 diabetes, the renal parenchyma would have to be responsive to insulin, unlike the liver which is insulin resistant. This was investigated by Feliers and colleagues; they employed a series of tests examining the status of insulin receptor activation and reported that kidney is responsive to insulin at the same time when liver is resistant to insulin in diabetic *db/db* mice [105]. These observations steered a series of investigations that identified a novel regulatory mechanism for ECM protein increment, mRNA translation [14, 15, 32], and also raised the possibility that hyperinsulinemia could participate in renal injury in type 2 diabetes.

There are two other models of type 2 diabetes which show progression of diabetic kidney disease that resemble human disease. The KKA^y^/Ta mice produced by transfection of the yellow obese gene (Ay) into KK/Ta mice are obese, diabetic mice that manifest hyperglycemia, hypertriglyceridemia, hyperinsulinemia, and microalbuminuria. KKAy mice developed hyperglycemia, hyperinsulinemia, and obesity after 16 weeks, with proteinuria, mesangial matrix accumulation, GBM thickening, and tubular dilation. It was considered a good animal model for the early pathology changes of DN [106–108]. The MKR mice which transgenically express mutant IGF-1R specifically in skeletal muscle develop insulin resistance in fat and liver with rapidly progressive beta-cell dysfunction and type 2 diabetes [109]. They exhibit early onset of the disease phenotype as seen by insulin resistance (as early as 4 weeks), fasting hyperglycemia (from 5 weeks), and abnormal glucose tolerance (at 7–12 weeks), and they develop kidney disease characterized by ECM accumulation [110].

## 8. Management of Diabetic Renal Disease

Currently available therapies are not completely effective in arresting progression of diabetic kidney disease, especially at more advanced stages of disease. Consistent with investigations discussed above [35], studies have shown that ACE inhibitors and ARBs are beneficial in reducing the progression of albuminuria in patients with type 2 diabetes [5, 72, 111]. Treatment with an ACE inhibitor has been shown to normalize expression of laminin in murine mesangial cells [112]. A recent report shows the use of antifibrogenic drugs that block TGF beta to be effective in restoring kidney function [113]. Although the angiotensin-converting enzyme inhibitors and angiotensin receptor blockers retard the progression of diabetic nephropathy, they are not able to halt the eventual development of end-stage renal disease [114, 115]. One reason could be that pathological changes in the kidney may already be in place preceding the clinical diagnosis of diabetic nephropathy owing to the cumulative effects of postprandial hyperglycemic excursions, metabolic syndrome, and insulin resistance in type 2 diabetes. We need to take into consideration that several pathologic processes work in consort to result in kidney injury in diabetes. To date the usual investigational approach has been linear, having adopted the traditional one-variable-at-a-time model. Future investigations should apply a systems biology approach to understand how multiple pathogenetic events occurring simultaneously result in renal injury in diabetes.

## 9. Conclusion

Deregulation of protein synthesis, processing, and degradation underlie the development of renal matrix changes induced by hyperglycemia in type 2 diabetes. Thus, attenuating ECM accumulation and/or enhancing ECM degradation is considered a prime target in the preventive treatment of diabetic renal complications. In order to achieve this objective identifying the molecular mechanisms by which high glucose stimulates matrix protein synthesis is of paramount importance. Understanding these mechanisms may help develop early detection strategies and help identify those subjects at risk of progressing to advanced kidney derangement. While optimal control of hyperglycemia is a highly desirable approach in the treatment of diabetic complications including nephropathy, the difficulty in achieving this goal due to inability to adhere to therapeutic regimens and adverse effects of intensive glucose control regimens require us to find additional therapeutic avenues. Such interventions can only be developed by truly understanding the pathogenesis of kidney injury in diabetes and identifying viable therapeutic targets.

## Abbreviations

ECM: Extracellular matrix protein
DN: Diabetic nephropathy
GBM: Glomerular basement membrane
TBM: Tubular basement membrane
4E-BP: 4E binding protein
AICAR: 5-aminoimidazole-4-carboxamide 1-*β*-ribofuranoside
AMPK: AMP-activated protein kinase
eEF: Eukaryotic elongation factor
eIF: Eukaryotic initiation factor
G*β*L: G protein*β*-subunit-like protein
GEC: Glomerular epithelial cells
TGF*β*: Transforming growth factor beta
IGF: Insulin-like growth factor
mTOR: Mammalian target of rapamycin
PTEN: Phosphatase and tensin homolog on chromosome ten
Raptor: Regulatory associated protein of TOR
Rheb: Ras homolog enriched in brain
TSC: Tuberous sclerosis complex
UTR: Untranslated region
UBF1: Upstream binding factor 1
VEGF: Vascular endothelial growth factor
PDCD4: Programmed cell death 4.
